# Macroprolactinemia in a Patient with Invasive Macroprolactinoma: A Case Report and Minireview

**DOI:** 10.1155/2013/634349

**Published:** 2013-01-15

**Authors:** Atanaska Elenkova, Zdravka Abadzhieva, Nikolai Genov, Vladimir Vasilev, Georgi Kirilov, Sabina Zacharieva

**Affiliations:** USHATE “Acad. Ivan Pentchev”, Clinical Centre of Endocrinology and Gerontology, Medical University-Sofia, 2 Zdrave Street, 1431 Sofia, Bulgaria

## Abstract

*Background*. Macroprolactin, the high-molecular prolactin isoform, is considered to be an inactive in vivo product with extrapituitary origin. Patients with macroprolactinemia are usually asymptomatic, with negative pituitary imaging. Based on these data, most authors do not recommend treatment and long-term followup in subjects with macroprolactinemia. However, there is evidence for overlapping clinical features among subjects with hyperprolactinemia due to monomeric or “big big” PRL isoform. *Case Presentation*. We present a 35-year-old female patient with secondary amenorrhea, mild obesity, hirsutism, headache and blurred vision. Hormonal evaluation revealed an extreme hyperprolactinemia (PRL = 10 610 mIU/L) almost exclusively due to macroprolactin isoform (MPRL = 10 107 mIU/L; recovery after PEG precipitation 4.7%) and hypogonadotropic hypogonadism. An invasive pituitary macroadenoma was visualized on MRI, and cabergoline therapy was initiated. Disappearance of clinical signs and symptoms, normalization of gonadotropin levels, and restoration of regular ovulatory menstrual cycles after 1 year of treatment are arguments in favor of preserved-macroprolactin bioactivity in this case. The significant decrease in MPRL levels and tumor volume in response to dopamine agonist therapy is suggestive for the tumoral origin of this isoform. *Conclusions*. Although macroprolactinemia is considered to be a benign condition, pituitary imaging, dopamine agonist treatment, and prolonged followup should be recommended in some particular cases.

## 1. Background

Three different isoforms of circulating human prolactin (PRL) have been identified: a monomeric (“little” PRL with a molecular weight of 23 kDa), a 50 kDa “big” PRL, and a high-molecular form (>l00 kDa), termed “big big” or macroprolactin [1, 2]. In physiological conditions the monomeric PRL accounts for 80–90%, the “big” PRL represents less than 10%, and macroprolactin (MPRL) represents a negligibly small percentage of the total PRL amount. Macroprolactinemia is a state of hyperprolactinemia characterized by predominant presence of the high-molecular PRL isoform in the circulation which has been considered biologically nonactive [3, 4]. On the other hand, there are published data about overlapping of the main hyperprolactinemia-related clinical symptoms in subjects with true hyperprolactinemia and those with macroprolactinemia [5–11]. This paper describes a case of invasive pituitary macroadenoma and secondary amenorrhoea with extremely elevated PRL levels almost exclusively due to macroprolactinemia successfully treated with dopamine agonist. A minireview of the literature on this topic is also included in the paper as a separate section.

## 2. Case Presentation

**Figure 1:**
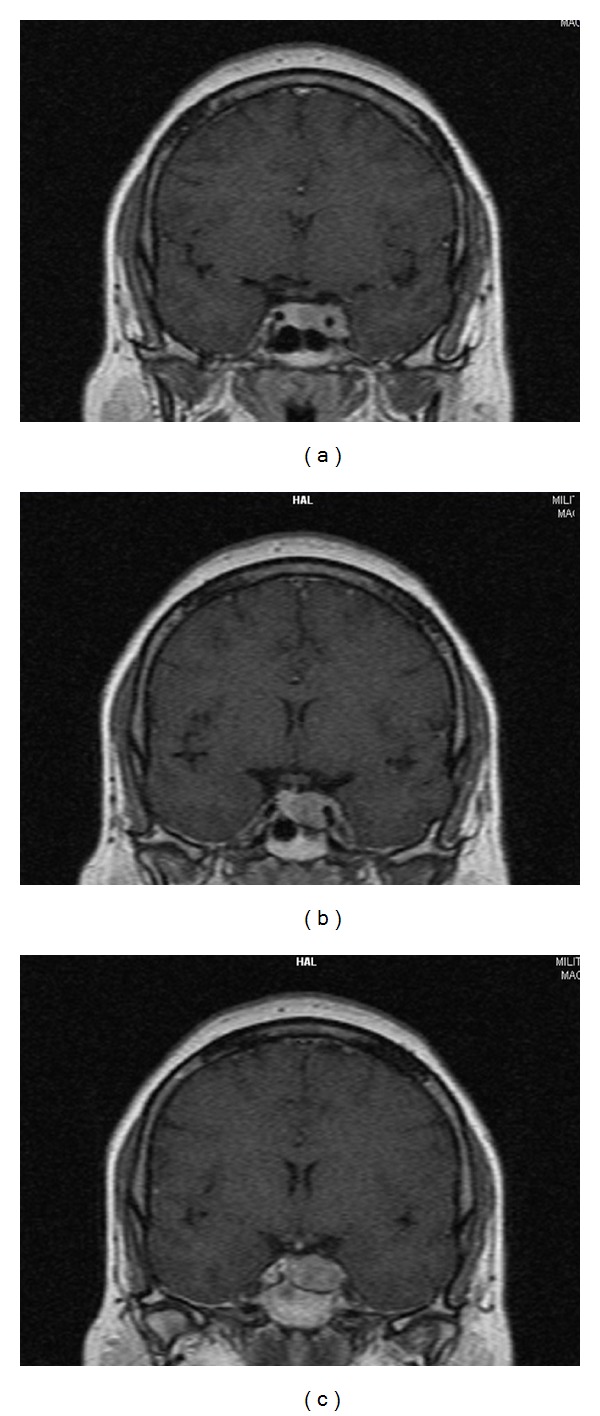
Postcontrast T1 weighted MRI at the time of diagnosis: coronal sections visualizing pituitary macroadenoma (21 × 13 mm) with the left cavernous sinus invasion.

**Figure 2:**
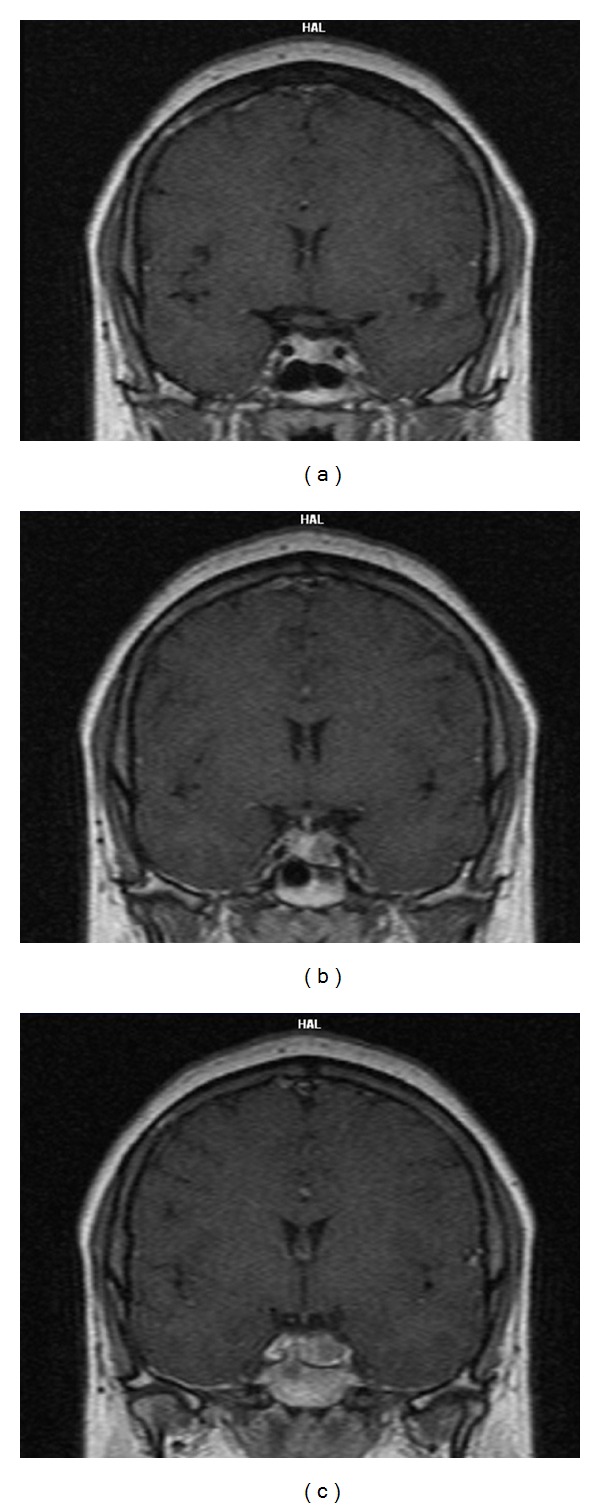
Postcontrast T1 weighted MRI after 1 year of cabergoline treatment: coronal sections at the levels corresponding to Figures 1(a), 1(b), and 1(c); a marked tumor shrinkage was seen (tumor size: 9 × 8 mm).

We present a 35-year-old female patient complaining of secondary amenorrhea, mild obesity, hirsutism, severe headache, and blurred vision. She had menarche at 15 followed by regular periods till the age of 20 when an oligomenorrhea occurred. After gynaecological consultation, treatment with Dydrogesterone (duphaston) was started using a standard dosage regimen: 10 mg daily from the 16th to the 25th day of the menstrual cycle. A good therapeutic response was initially achieved, and the patient could maintain regular menstrual cycle for about one year. After that, recurrence of oligomenorrhea occurred in spite of the twofold increase of Didrogesterone dose. The treatment was discontinued which resulted in a permanent amenorrhea. The patient was referred to the University Hospital of Endocrinology where full clinical and hormonal evaluations were carried out. *Physical examination* revealed mild hirsutism (Ferriman-Gallwey score  =  13), visceral obesity (BMI  =  31.2%), and no galactorrhea. *Hormonal analysis:* Venous blood samples were taken in the morning, after 30 minutes of rest (sampling referred to the second and third admissions made during the early follicular phase of the menstrual cycle) (Table 1). Serum PRL, E2, LH, FSH, and Testosterone (T) levels were measured by the use of commercially available kits “Immunotech” (Beckman-Coulter, France) with analytical sensitivity: for PRL < 14.5 mIU/L; for LH and FSH < 0.2 IU/L; for E2 < 22.02 pmol/L; and for T < 0.087 nmol/L, respectively; reference ranges (female subjects): PRL < 550 mIU/L; LH (follicular phase): 2,0–10,0 U/L; FSH (follicular phase): 1,0–10,0 U/L; E2 (follicular phase): 90–550 pmol/L; T: 0.3–3.5 nmol/L. A sensitive immunoradiometric assay (intra- and interassay CV: 2.8% and 8%, resp.,) was used for PRL detection in which concentrations were determined twice, in the serum immediately after thawing and in the supernatant after precipitation with polyethylene glycol (PEG 8000). Percentage of macroprolactin (MPRL) was calculated using the following formula: MPRL% = (PRL serum − PRL  supernatant) × 100/PRL serum. Results of the PEG precipitation test were presented as a recovery % (free PRL) = 100% − MPRL%. Macroprolactinemia was considered present when a recovery % was <40%, and monomeric PRL levels after PEG treatment were within the normal range [12–15]. Hormonal analysis showed normal thyroid function (TSH  =  0.85 mIU/L; FT4  =  10.3 pmol/L), testosterone levels (T  =  2.4 nmol/L), extremely high serum PRL levels (PRL  =  10610 mIU/L), and suppressed gonadotropins levels (LH  =  1,1 U/L; FSH  =  1,2 U/L; E2  =  235 pmol/L). PEG precipitation test revealed that hyperprolactinemia was almost exclusive due to the presence of the high-molecular prolactin isoform (MPRL  =  10 107 mIU/L; recovery  =  4,7%). *Instrumental evaluation:* Ovulatory function assessment was based on transvaginal ultrasound folliculometry performed by a single experienced gynecologist using Toshiba Eccocee (SSA-340A) Ultrasound Machine. Repeated exams demonstrated severely impaired folliculogenesis and anovulation. *Contrast-enhanced high-resolution magnetic resonance imaging (MRI)* revealed an invasive macroadenoma (21  ×  13 mm) with left cavernous sinus involvement (left internal carotid artery encasement) and no evidence of optic chiasm compression (Figures 1(a), 1(b), and 1(c)). *Ophthalmological examination* did not detect any visual field deficits. *Treatment:* Cabergoline treatment was initiated at a dose of 2.0 mg per week. *Follow-up:* Stable normoprolactinemia and complete disappearance of clinical symptoms with normalization of menstrual cycle were achieved after one year of cabergoline therapy (2 mg/week; cumulative dose  =  96 mg). Restoration of ovulation was objectified by transvaginal ultrasound folliculometry. Significant weight loss (7 kg; 8.2%; BMI  =  28.6 kg/m^2^) and beneficial effect of dopamine agonist treatment on the headache and general condition were also reported by the patient (Table 1). A marked reduction in the tumour size was registered on MRI after 1 year of cabergoline treatment (Figures 2(a), 2(b), and 2(c)).

## 3. Minireview of the Literature

Issues regarding biological activity and the site of macroprolactin synthesis are still not completely resolved. According to the literature the majority of patients with macroprolactinemia are oligo- or asymptomatic which supports the hypothesis for a decreased biological activity of the high molecular prolactin isoform [3, 4, 16]. On the other hand, there are enough publications about overlapping of the main hyperprolactinemia-related clinical symptoms (oligomenorrhea, galactorrhea, etc.) in subjects with monomeric hyperprolactinemia and those with macroprolactinemia [5–11, 17]. This controversy could be explained by heterogeneity of the MPRL structure. In the majority of cases, the high molecular form consists of complexes of PRL and anti-PRL autoantibodies predominantly of IgG class, specific to human PRL with a low affinity and high capacity [18–20]. Rarely, MPRL exists as complexes of PRL with IgA and IgM or aggregates of covalent or noncovalent polymers of monomeric PRL [2, 21]. Macroprolactin consisted of PRL-IgG complexes has been shown to exert a full bioactivity in vitro but a decreased bioactivity in vivo which is considered to be a result from the lower bioavailability due to impaired transendothelial transfer of high molecular complexes [13, 19, 22, 23].

Macroprolactin synthesis is thought to be an extrapituitary postsecretory phenomenon [13, 15, 24]. On the other hand, pituitary adenomas are revealed in about one fourth of patients with macroprolactinemia [10, 25, 26]. One recent study based on MRI with postcontrast enhancement has even shown pathological pituitary scans in 44% of macroprolactinemic subjects [5]. Two explanations are possible: a coexistence of pituitary adenoma and macroprolactinemia or macroprolactin production by pituitary tumor itself. Literature data have provided evidence in favor of both possibilities. Leslie et al. have shown a normal chromatographic pattern with predominance of the monomeric prolactin isoform in tissue samples from pituitary adenomas in patients with macroprolactinemia—an argument supporting the hypothesis of peripheral mechanism of macroprolactin synthesis [25]. In contrast, two studies have demonstrated significantly higher concentrations of big-big PRL in extracts from prolactinoma tissue compared to those in samples from normal adenohypophysis [27, 28]. Coexistence of pituitary nonfunctioning adenoma and macroprolactinemia could be suggested in patients with oligosymptomatic clinical presentation. To date, only one case report has been published in support of the thesis for the tumoral origin of MPRL. Lakatos et al. presented the history of a 80-year-old man with an intra- and parasellar pituitary tumor (21  ×  12 mm in size) and marked hyperprolactinemia mainly due to macroprolactinemia (PRL total 514 ng/mL; MPRL 436 ng/mL; recovery 15,2%). The patient had mild subclinical primary hypothyroidism, low-normal gonadotropines, decreased testosterone levels, no hypocorticism, and growth hormone deficiency. Stable normoprolactinemia and a remarkable shrinkage of the pituitary tumor were achieved after 9 months of dopamine agonist treatment (Quinagolide). Based on this good therapeutic response the authors suggested tumoral origin of macroprolactin in this patient [6]. Nevertheless, this case does not provide strong evidence for the biological activity of macroprolactin. On one side, the majority of male patients with prolactinomas have an oligosymptomatic presentation with a decreased libido as the most common manifestation. On the other, the hypogonadotropic hypogonadism in this particular case could be a result not only of the marked hyperprolactinemia, but also of the tumor mass effect. Moreover, the patient's age cannot be excluded as an additional factor for the low testosterone levels.

## 4. Discussion

Our female patient with invasive macroprolactinoma and proven macroprolactinemia with extremely high PRL levels and typical clinical presentation represents an example that in some although very rare cases the high-molecular PRL isoform may exert preserved biological activity. Restoration of regular ovulatory menstrual cycle under dopamine agonist treatment supports this hypothesis. The decrease of MPRL levels after dopamine agonist treatment could suggest a tumoral origin in this case. Marked reduction in the pituitary tumour volume well corresponding with the clinical and laboratory improvement is another strong argument in favour of this thesis.

## 5. Conclusions

In the vast majority of cases, although long-lasting and relatively stable, macroprolactinemia is associated with mildly elevated prolactin levels, oligo- or asymptomatic presentation, and negative pituitary imaging and does not need further investigation and treatment. Macroprolactinemia associated with invasive pituitary prolactinoma presenting with hyperprolactinemia-related clinical manifestations is an extremely rare condition which requires long-term therapy and followup. Similar to monomeric hyperprolactinemia, conservative treatment with dopamine agonists seems to be safe and effective, thus preventing unnecessary transsphenoidal surgery and possible complications in these particular cases.

## Figures and Tables

**Table 1:** Main clinical, laboratory, and instrumental findings.

Parameters	35-year-old female patient	
	Baseline	1-Year Follow-up^1^
Prolactin, PRL (mIU/mL)	10 610	295
Macroprolactin, MPRL (mIU/mL)	10 107	106
Recovery, %	4.7%	36%
Tumor size (mm) (on MRI)	21 × 13 mm	9 × 8 mm
Body mass index, BMI (kg/m^2^)	31.2%	28.6%
Main patient's complaints	Severe headache; blurred vision	No headache; normal vision
Hirsutism, Ferriman-Gallwey score	13	13
Menstrual cycle	Secondary amenorrhea	Regular
Pelvic ultrasound	Anovulation	Normal

^1^Treatment with Cabergoline 2.0 mg/week; cumulative dose = 96 mg.

## Abbreviations

PRL: Prolactin
MPRL: Macroprolactin
BMI: Body mass index
E2: Estradiol
LH: Luteinizing hormone
FSH: Follicle stimulating hormone
T: Testosterone
PEG: Polyethylene glycol
TSH: Thyroid stimulating hormone
FT4: Free thyroxin
MRI: Magnetic resonance imaging.
